# Editorial Comments to the Special Issue: “*Legionella* Contamination in Water Environment”

**DOI:** 10.3390/pathogens9121017

**Published:** 2020-12-02

**Authors:** Silvia Bonetta, Sara Bonetta

**Affiliations:** 1 Department of Life Sciences and Systems Biology, University of Torino, Via Accademia Albertina 13, 10123 Torino, Italy; 2 Department of Public Health and Pediatrics, University of Torino, Piazza Polonia 94, 10126 Torino, Italy

## 1. Introduction

*Legionella* spp. are ubiquitous microorganisms that are widely distributed in aquatic environments. From these natural reservoirs, this opportunistic pathogen can spread to and colonize artificial aquatic environments [1]. Water systems of large buildings, such as hospitals, thermal baths, hotels, and dental units are often contaminated by legionellae [2,3] and various parameters such as physical, chemical, and microbial building water system characteristics can influence *Legionella* occurrence [4]. *Legionella* are intracellular bacteria whose natural hosts are aquatic protozoa in which these bacteria replicate and are protected from harsh environmental conditions [2].

*Legionella pneumophila* is most frequently associated with human disease (Legionnaire’s disease-LD or Pontiac fever); however, other species, including *L. bozemanae*, *L. dumoffii*, and *L. longbeachae* also cause human infections. The most common way of contagion is via aerosols inhalation containing infectious *Legionella* from showerheads, certain medical equipment (e.g., respiratory equipment), cooling towers, hydrotherapy equipment, and decorative fountains [5].

A range of physical and chemical disinfection methods have been proposed with the aim of controlling *Legionella* contamination; however, to date, the most effective procedures have not been defined [6,7]. Therefore, alternative disinfection methods that are effective in controlling the proliferation of *Legionella* could be useful tools to reduce the risk of the spread of Legionnaires’ disease.

Surveying and monitoring of legionellae in water systems is needed for risk assessment and prevention of legionellosis. However, although the assessment of *L. pneumophila* in water is typically performed by culture isolation on selective media, it has several limits including the long incubation times and the inability to detect the viable but non-culturable bacteria (VBNC). For this reason, in the last decades, alternative tools for rapid, sensitive, and specific detection of *Legionella* in water samples have been proposed [7,8]. For the identification of possible sources of contamination/infection, high-resolution genotyping of new isolates (e.g., Sequence Based Typing, Multilocus Variable number of tandem repeats) is needed to correlate environmental with clinical isolates.

In order to increase the knowledge on different aspects of *Legionella* contamination in the water environment, this Special Issue aims to bring together research studies related to the occurrence of *Legionella* in water systems of different critical environments (hospital, hotel, large buildings); the role of different factors that can influence the *Legionella* contamination (e.g., disinfection treatment, water characteristics, plumbing materials, and protozoa presence) as well as the advantages and disadvantages of different methodological approaches were also addressed.

## 2. Occurrence of *Legionella* in Large Building Water Systems with Different Methodological Approaches

Many studies have demonstrated that the main sources for LD are the drinking water distribution systems (DWDS) in large buildings such as hospitals and hotels. In particular, the *Legionella* contamination of hospital water systems posed a high risk for patients and hospital staff. The study of Zayed and collaborators [9] showed a different distribution of the *L. pneumophila* population in DWDS of eight hospitals throughout the West Bank, highlighting a low concentration of culturable *L. pneumophila* in water, but a higher prevalence in biofilm. The detection method used influenced the results obtained: in fact, PCR analyses showed a higher detection rate in water and biofilm with respect to culture analyses. The study of environmental isolates is needed for the characterization of the *Legionella* population and to identify possible sources of infection. The genotyping with Multilocus Variable number of tandem repeats Analysis using 13 loci (MLVA-8(12)) identified 20 genotypes only described for the West Bank and they were attributed to individual groundwater based water supplies. The comparison between the MLVA genotyping and the standard Sequence Based Typing (SBT) methods showed that MLVA was highly consistent with SBT, but showed a higher resolution. This method provides a good basis for detailed studies of the health and water management relevant traits of *L. pneumophila* in support of a better clinical and DWDS management. The Whole Genome Sequencing (WGS) also provides detailed genetic information about the *Legionella* strain in large building water system, beyond that obtained from SBT alone, enabling potential subspecies identification, refined taxonomic classification, and genetic profiling for virulence properties [10].

Different studies showed variable rates of contamination and species diversity of *Legionella* in water systems of other large buildings such as hotels, in non-outbreak situation. However, only little information was available on the molecular diversity of *Legionella* spp. in hotel settings. In the study of Yakunin and collaborators [11], the results obtained in Israeli Hotels highlighted a relevant *Legionella* contamination of the DWDS. In 37% of the investigated hotels, *Legionella* spp. counts exceeded the regulatory threshold (1000 CFU/L). The most frequently contaminated water sources were cooling towers followed by faucet, hot tubes, water lines, and storage tanks. In the same study, as also reported by Zayed et al. [9], several *Legionella* strains were found to be related to specific geographical regions. This finding could be associated with the water differences between the regions, i.e., physical and chemical properties. The results obtained highlighted the importance of investigating the prevalence and diversity of *Legionella* strains in hotel buildings in different geographical regions in order to facilitate the risk assessment, surveillance, and control measures of travel-associated Legionnaire’s disease.

## 3. Prevention and Control of *Legionella* Contamination

The new revision of the European Drinking Water Directive, such as the WHO guidelines for Drinking Water Quality, suggests the approach of the Water Safety Plan to evaluate the risk associated to the main pathogens involved in waterborne diseases including *Legionella*. The Italian Guidelines support the development of a risk assessment plan and emphasize the need for an adequate environmental surveillance plan. The study of Mazzotta et al. [12] highlighted, during a *Legionella* environmental Surveillance Program in different hospitals, the critical role of Surgical and Washing Outlets (SHWO) with Thermostatic Mixer Valves (TMV) in bacterial growth and Health Care-Associated Infections (HAIs) risk. A non significant difference of *Legionella* contamination between hot and cold samples demonstrated a continuous mixing between two pipelines that create an environment capable of supporting *Legionella* growth. The characteristics of the mixed water produced are also able to influence the distribution of isolates (*L. pneumophila* percentage > in hot water). The results obtained underlined the importance of the implementation of environmental surveillance programs with the aim to deepen the critical points.

A wide variety of disinfection techniques (e.g., chemical disinfection, UV, high temperature) can be used for the prevention and control of *Legionella* contamination in the water network. Girolamini et al. [13] have evaluated that the long term H_2_O_2_/Ag^+^ treatment, a low cost disinfectant easy to dose and not very aggressive on the pipelines, is a good strategy to decrease risk in the hospital, reducing the *Legionella* contamination level. However, to guarantee the efficiency of the *Legionella* reduction, it is also necessary to consider the building characteristics, apply an adequate risk assessment plan, increase the monitoring samples size and regulate the dosage in relation to the *Legionella* loads. These infection prevention strategies can be applied to reduce the risk that is also coming from other contaminated healthcare facilities, such as Dental Unit Water Lines (DUWLs), as reported by Tuvo et al. [14]. In this work, the authors highlighted that an implemented risk management plan, that include filters installation and shock disinfection with a solution of 4% hydrogen peroxide and surfactants, appears to be a promising alternative for decreasing *Legionella* colonization in DUWLs of Hospital Clinics. In this context, it is important to highlight that, in the dental unit investigated, a water safety plan, a maintenance plan, and a control program were constantly applied, but there was a low adherence to good practices in DU management. A low adherence to the best practice guidance had probably contributed to biofilm proliferation, making necessary measures that are more restrictive.

## 4. Parameters Influencing the *Legionella* Occurrence in Building Water Systems

Various parameters such as physical (temperature, pH range, hardness), chemical (disinfectant, pipe materials), microbial (free-living amoeba, protozoa), and characteristics of building water systems can influence *Legionella* occurrence. In their paper in the present Special Issue, Cullom and co-workers [15] systematically reviewed the literature to critically examine the varied effects of common metallic (copper, iron) and plastic (PVC, PEX) pipe materials on factors influencing opportunistic pathogens such as *Legionella* growth in drinking water, including the nutrient availability, disinfectant levels, and the composition of the broader microbiome. Plastic pipes demonstrate a lower disinfectant demand while iron pipes exhibit a high disinfectant demand and they can favor the biofilm colonization. Although copper pipes are known for their antimicrobial properties, under some circumstances, copper’s interactions with premise plumbing water chemistry and resident microbes can encourage growth of opportunistic pathogens. Plumbing design, configuration, and operation can be manipulated to control such interactions and health outcomes. The influences of pipe materials on opportunistic pathogen physiology should also be considered, including the possibility of influencing virulence and antibiotic resistance. Moreover, the study of Martin and co-workers [16] demonstrated, under controlled laboratory conditions, the importance of considering interactive effects with flow and pipe materials, particularly with respect to relative water corrosivity and influence on residual chlorine levels, in keeping *Legionella* levels low. The complex interaction between the various chemical, physical, and microbiological parameters and *Legionella* contamination is also highlighted by Buse et al. [10]. In their study, negative and positive correlations between *Legionella* and some water characteristics (pH, temperature, turbidity, chlorine, Heterotrophic plate count, and *Vermamoeba vermiformis* contamination) were observed and they varied between location and sample types. The authors concluded that future studies would help elucidate ways to effectively manage the risks associated with *Legionella* exposure within the drinking water distribution systems.

The relationship between *L. pneumophila* contamination and environmental drivers (e.g., temperature, pH, conductivity, iron, nitrate, nitrite, ammonia, copper, phosphate, zinc, hardness, magnesium, calcium of bulk water) was also investigated by Zayed et al. [17]. Statistical analyses with physico-chemical parameters revealed a decrease of *L. pneumophila* abundance in water and biofilms with increasing magnesium concentrations. MLVA-genotype analysis of the *L. pneumophila* isolates and their spatial distribution indicated three niches characterized by distinct physico-chemical parameters and inhabited by specific consortia of genotypes. This study provides novel insights into mechanisms shaping *L. pneumophila* populations and triggering their abundance leading to an understanding of their genotype-specific niches and ecology in support of improved prevention measures.

Some water quality measurements have been suggested as alternative approaches to predict the *Legionella* risk for building’s water system instead of directly culturing analysis. However, as reported in the study of Pierre and collaborators [18], a poor correlation and a low positive predictive value between the hot water return line and distal outlet positivity in different buildings was revealed. Moreover, no correlation between *Legionella* distal site positivity and total bacteria, pH, free chlorine, calcium, magnesium, zinc, manganese, copper, temperature, total organic carbon, or incoming cold-water chlorine concentration was observed. These data confirm that these water quality parameters should not be used alone to determine the building’s *Legionella* colonization rate and effectiveness of water management programs.

## 5. Sensitivity and Selectivity of Different Culture Media for *Legionella* Detection

The plate culture method using specific media usually supplemented with different combinations of antimicrobial selective substances is considered the gold standard for the detection and enumeration of *Legionella* in water samples. The culture method is generally performed according to standards, such as the International Standard Organization (ISO); in particular, the ISO 11731 is the most used and it has recently been updated. These updates introduced the utilization of different media: (I) the buffered charcoal yeast extract (BCYE) agar, (II) the BCYE with selective supplements (BCYE+AB) containing polymixin B, sodium cefazolin and pimaricin, (III) the highly selective Modified Wadowsky Yee (MWY) agar or, as an alternative, the glycine, vancomycin, polymyxin B, cycloheximide (GVPC) agar. The MWY is the best medium for isolating *L. pneumophila* from potable water samples, while GVPC was proposed in water samples characterized by high interfering microbial flora. Despite these premises, the study conducted by Scaturro et al. [19] in potable water samples with low interfering microorganisms observed that GVPC was more efficient in detecting *Legionella* contamination than the BCYE medium. Moreover, no significant difference of *Legionella* loads (CFU/L) was found between BCYE and GVPC agar plates. Furthermore, the possibility of improving the isolation of *Legionella* non-*pneumophila* species on BCYE was not confirmed. These results make questionable the need to utilized BCYE agar plates to analyze potable water samples.

Given that the recovery of *Legionella* spp. strictly depends on the type of agar being used, quality-assured culture media for water testing are key to consumer safety. In this context, Ditommaso and collaborators [20] reported a comparative assessment of the sensitivity and selectivity of MWY and BCYEα media supplied by two different manufacturers (Xebios Diagnostics and Oxoid) in water samples. Even though the analysis showed an excellent agreement between the recovery rates of the four media tested, the quantitative recovery of *Legionella* spp. colonies using Xebios media was significantly greater than that achieved by Oxoid media. Furthermore, the sensitivity of detection was significantly higher when samples were plated on MWY Xebios agar, while the selectivity of MWY appeared to be the same regardless of the manufacturer. Finally, the MWY Xebios medium enhanced the recovery of non-*pneumophila Legionella* species. The results obtained confirmed that culture protocol standardization, as well as quality control of the culture media, are essential to achieve intra- and interlaboratory reproducibility and accuracy.

Although plate culture methods are the gold standard for *Legionella* detection in water samples, they have high variability in the enumeration, are time consuming, and require significant experience in recognizing *Legionella* colonies. A promising alternative method is the Legiolert test, a liquid culture method based on bacterial enzyme detection technology, which determines the most probable number (MPN) of *L. pneumophila* species in water samples. Scaturro et al. [21] highlighted that the plate culture method (MWY) and Legiolert method were comparable and concluded that Legiolert may be considered as a valuable test for the detection and enumeration of *L. pneumophila* in potable water samples and it can be used as a valid alternative to the traditional plate culture methods.

## 6. The Role of Protozoa in the *Legionella* Contamination in Water Distribution System

The implication of interaction with a protozoan host for the control of *L. pneumophila* as well as the efficacy of potable water disinfection protocols on *L. pneumophila* and host protozoan are essential and they were reviewed by Nisar and collaborators [22]. The systematic review highlights that protozoan hosts facilitate the intracellular replication and packaging of viable *L. pneumophila* in infectious vesicles, while cyst-forming protozoans provide protection from prolonged environmental stress. Moreover, the data collected underline the failure of common disinfection procedures to achieve long-term elimination of *L. pneumophila* and protozoan hosts from potable water. This overview report that the disinfection procedures and protozoan hosts also facilitate biogenesis of viable but non-culturable (VBNC) *L. pneumophila*, which have been shown to be highly resistant to many water disinfection protocols. However, other studies have demonstrated that all free-living amoebae (FLA) do not exhibit the same behavior when they are exposed to *L. pneumophila* strains. In fact, the *Villaertia magna* strain C2c Maki has been demonstrated to eliminate the *L. pneumophila* serogroup 1 strain Paris. The results obtained in the study of Hasni et al. [23] confirmed that none of the *Legionella* strains tested (Paris, Philadelphia, and Lens) exhibit intracellular growth and that the *V. magna* strain C2c Maki decreases the number of internalized *L. pneumophila.* Thus, these results support the idea that the *V. magna* strain C2c Maki has a unique behavior in regard to *L. pneumophila* strains. The non-permissiveness of *V. magna* C2c Maki toward *L. pneumophila* strains was confirmed by Mameri et al. [24]. This study demonstrated that *V. magna* C2c Maki did not increase the expression of different virulence genes (htpX, icmE, lirR, ccmF, gacA, tatB, and lvrE) of *L. pneumophila* strains in contrast to *Acanthamoeba castellanii*.
